# Supplementary material for: Are carbon emissions trading and green financial instruments synergistic? -Comprehensive quantitative research based on content analysis
Source: PLoS One. 2024 Mar 7;19(3):e0298601. doi: 10.1371/journal.pone.0298601 (PMC10919601; doi:10.1371/journal.pone.0298601)
Supplement: S1 Data — (ZIP) [file pone.0298601.s001.zip › Data/地级市Z_F工作报告.zip!/╦─┤¿/╣π╘¬╩╨/└┤╫╘╣1⁄2╓┌║┼í╛┬φ┐╦╩2╛▌═°í┐_2014.docx]

各位代表：

　　现在，我代表市人民政府向大会报告工作，请予审查，并请市政协委员提出意见。

2013年工作回顾

　　2013年，在省委、省政府和市委的坚强领导下，在市人大和市政协的监督支持下，市政府认真履行职能，紧密团结和依靠全市人民,全力以赴稳增长，加大力度调结构，求实创新促改革，多措并举惠民生，全市经济社会发展呈现出“稳中有进、持续向好”的良好局面。全市实现地区生产总值518.75亿元，增长10.5%，增速居全省第11位，比上年提升2位；地方公共财政收入30.46亿元，增长13.5%；城镇居民人均可支配收入18713元，增长10%；农民人均纯收入6442元，增长14%，增速居全省第4位。

　　一、投资消费平稳增长，经济发展后劲逐步增强

　　项目投资稳中有增。创新项目推进工作机制，切实加强土地、资金等要素保障，着力建设以大项目带动大投入、培育大产业、推动大发展的良好环境。完成全社会固定资产投资541.09亿元，增长5%。长虹200万台电视机整机、兴能新材料锂电池及正负极材料等重点产业项目竣工投产，亭子口水利枢纽工程实现两台机组并网发电，广英服装、中纺集团60万吨油脂加工等一批重点产业项目开工建设，兰渝铁路、西成客专等重大项目顺利推进。

　　投资结构逐步优化。171个市列重点项目完成投资324.8亿元，占年度目标的116.9%。重点领域投资实现较快增长，交通运输业投资106.99亿元，增长11.5%；水利、环境和公共设施投资72.2亿元，增长10.9%；工业投资179.4亿元，增长4.1%。认真落实鼓励民间投资的各项政策措施，民间投资274.87亿元，增长3%，投资额占全市固定资产投资的50.8%。

　　消费需求持续扩大。积极推进城乡现代流通体系建设，加快农贸市场改造升级，扎实开展秦巴山区年货节等各类促销活动，大力促进旅游休闲、文化教育、家政养老等服务消费，继续实施农机购置补贴，合理引导住房、汽车等消费。社会消费品零售总额实现219.46亿元，增长13.7%。加强价格调控监管，居民消费价格指数涨幅2.9%。

　　二、产业发展稳步推进，转型升级取得新进展

　　工业经济较快增长。实现全部工业增加值215.07亿元，增长13.7%。实现规模以上工业增加值194.6亿元，增长13.1%，增速居全省第3位，比上年提升1位。“5+2+1”产业体系初步形成，食品饮料、建材、能源化工、战略性新兴产业产值均突破100亿元。下决心转方式调结构，淘汰落后产能企业31户，关闭小煤矿26家。新增园区面积10.95平方公里，新建标准化厂房12.88万平方米，剑阁经济开发区成功创建省级经济开发区。新培育规模以上企业62户。新培育国家高新技术企业7户，成功申报国家地理标志保护产品5个，新创建四川名牌24个，新认定四川省著名商标4件。

　　现代农业提质增效。实现农业增加值94.31亿元，增长3.6%。粮油生产实现“七连增”。新建万亩现代农业园区10个。新建特色产业化标准基地48.1万亩、土鸡生态养殖小区50个。新型农业经营体系建设加快推进，新培育农业产业化龙头企业42家，新发展农民专合组织341个，其中省级22个、居全省第2位，新发展家庭农场220家、专业大户4586户。苍溪乐园、青川曲河、朝天双峡湖等水库开工建设，亭子口灌区一期等重点水利工程有序推进。“五个三”农田水利基本建设经验、特色产业扶贫助农增收经验在全省推广。

　　服务业发展态势良好。实现旅游总收入112.6亿元，增长35.8%。成功创建省旅游标准化示范市，剑门关─翠云廊创国家5A级旅游景区通过省检，青溪古镇成功创建国家4A级旅游景区。蜀汉文化产业园评为首批省级文化产业园，苍溪县建成全国休闲农业与乡村旅游示范县，昭化区荣获“美丽中国”十佳旅游县(区)称号。引进川北国际物流港、远成物流等重点物流项目并顺利推进，万达广场、老城中央商务区等城区四大商圈和25条特色街区加快建设，网购网销广元专区平台注册企业达1120家，建成农村社区综合服务中心380个。金融服务业健康发展，年末银行业金融机构各项存款余额935.51亿元，增长12.2%；各项贷款余额415.74亿元，增长24.3%；保险业保费收入21.54亿元，增长11.5%；证券交易额154.9亿元，增长35.9%。

　　三、统筹发展加快推进，城乡面貌进一步改善

　　新型城镇化步伐加快。新增建成区面积10平方公里，城镇化率提高1.38个百分点。大力推进万源新区、三江新区建设，广元火车站广场主体工程完工，瓷莲路、老城人防工程、市城区雨污分流工程等市政项目加快建设，中心城区城市品位不断提升。县域城镇建设加快推进，城镇功能进一步完善。积极参加全省“百镇建设试点行动”，羊木等5个试点镇完成总体规划编制。加快27个重点镇基础设施建设，引导各类要素向城镇集聚，建成城乡新型社区617个。城乡环境综合治理深入推进，城市管理水平不断提高，创建国家卫生城市通过省级考核评估。

　　幸福美丽新村加快建设。新建各类新村聚居点87个，新建生态小康新村200个。新建通村公路1600公里，改造农村电网79千米，建成农村供水工程1400处，公共服务设施不断完善。新农村建设规划布局、连片推进等做法得到省委、省政府主要领导肯定，3个村荣获全国首批美丽乡村称号。

　　生态文明建设有力推进。全面启动低碳城市试点，扎实推进低碳示范社区、低碳工业示范园区创建工作，创建国家新能源示范城市获国家能源局批准。新建农户沼气池1.94万口，建成省级沼气化市，完成农村户用清洁能源改造1.3万户，森林覆盖率54.3%，成功创建国家森林城市。建成主城区绿道22公里，南河湿地公园荣获全省首个国家湿地公园称号，红星公园等评为首批省重点公园。创建成省级生态乡镇23个、省级园林新村2个。省环保模范城市创建工作加快推进。节能减排目标全面完成，全市万元生产总值和工业增加值能耗分别下降5.39%、8.1%，化学需氧量、氨氮、二氧化硫、氮氧化物减排均超额完成省定控制目标。全市饮用水源地水质全部达标,城市空气环境优良天数达95%以上，环境空气质量持续居全省前3位。

　　四、改革开放有序推进，发展活力不断增强

　　重点领域改革不断深化。继续深化统筹城乡综合配套改革，农村土地承包经营权确权登记工作进入航拍实测阶段，集体林权、集体土地使用权、户籍制度、农村金融等改革全面深化，利州区农村改革综合实验区试点成效显著。深化行政管理体制、行政审批制度、行政权力清理规范和事业单位分类改革，完成食品药品监管机构改革，新设基层监管所48个。城市管理相对集中行政处罚权工作全面开展。深化财税管理改革，加强全口径财政资源统筹力度，“四本预算”全部纳入预算管理，出台加强BT项目监管规定，防控政府债务风险，财政资金使用绩效明显提升，“营改增”试点工作顺利开展。医药卫生体制改革目标如期实现，14家县级公立医院全面停止药品加成。市属国有企业建立现代企业制度改革迈出新步伐，广运集团上市进入全面辅导期。民营经济快速发展，实现增加值280.17亿元，增长12.3%，占全市生产总值的54%。

　　开放合作水平全面提升。全面促进对外开放，到位市外资金378.03亿元，增长15.5%，成功引进万达广场、新中方健康产业城等一批大项目。积极参加老促会支援老区建设、央企入川、港澳企业四川行、民企入川、华商大会、西博会等重大投资促进活动，成功承办第六届中国广元国际中小企业交易会，浙广合作、九广合作、厅市合作、市校合作全面深化。

　　五、社会事业全面发展，公共服务能力明显提升

　　科技创新取得积极进展。加强科技工作，市和利州区首次荣获全国科技进步先进市、区称号。飞亚新材料技术中心创建成全市首个国家级企业工程技术研究中心，创建成省级重点实验室、省级企业工程技术研究中心2个，建成市级院士（专家）工作站、重点实验室等研发平台18个，取得重大科技成果31项，科技成果转化产值同比增长25%。专利申请723件，实施专利转化项目128项，专利产品实现产值14.5亿元。创新开展科普工作，首次获得全国科普活动优秀组织奖。

　　教育事业协调发展。推进义务教育均衡发展，学生营养改善计划惠及19.52万农村义务教育阶段学生，其经验在全国推广。“学前教育三年行动计划”基本完成，学前教育三年毛入园率达80%，高于全省平均水平。实施创新拔尖人才培养计划，高考本科上线稳定在万人以上。职业教育重点专业建设切实加强，校企合作全面深化，高等教育发展势头良好，民办教育进一步规范，残疾少年儿童入学率达到80%，“9+3”免费教育工作受到省政府表彰。

　　卫生事业健康发展。卫生服务体系逐步完善，综合服务能力全面提升，新建省标准化乡镇卫生院127所、村卫生室1197个。新农合参合率达98%以上，困难群众特大疾病医疗救助覆盖率100%，城乡居民健康档案规范化电子建档率96.15%。基本药物制度全面落实，重大疾病防控工作切实加强，孕产妇和婴儿死亡率分别降至13.46/10万、5.52‰。

　　文化体育事业繁荣发展。市文化艺术中心全面建成并对外开放，县区文化馆（图书馆）、乡镇综合文化站、农村书屋覆盖率100%，村级文化活动室覆盖率85%，建成示范乡镇综合文化站23个。成功举办女儿节，成功承办全省第十四届戏剧小品（小戏）大赛。文艺创作成效明显，舞蹈《幸福像花儿一样》荣获全国群众文化最高政府奖项─第十六届“群星奖”。电视节目质量不断提升，广播电视综合覆盖率98.13%。成功举办首届市运会，群众体育蓬勃发展。

　　其他工作取得新进展。持续稳定低生育水平，人口自然增长率3.26‰。加强食品药品安全监管，连续六年实现“零事故”。防灾减灾综合能力大力提升，朝天区被评为全国首批五个防震减灾示范县（区）之一。审计质量有效提升，现代化服务型统计建设不断加强。苍溪、旺苍、朝天档案馆成功创建国家级综合馆。人防、测绘地理信息、气象、消防、科普、工会、妇女儿童、青少年、老龄、残疾人、保密、地方志、红十字会、慈善和民族宗教等工作都取得了新成绩。

　　六、民生持续有效改善，社会大局保持和谐稳定

　　民生持续有效改善。全市财政民生支出120.5亿元，占财政支出的68%，“十项民生工程”全面完成。省级创业型城市工作全面启动，新增城镇就业3.94万人，城镇登记失业率3.89%。转移农村劳动力就业96.98万人，实现劳务收入123.96亿元。社会保障体系日益完善，各项社会保险累计实现参保265.29万人次。开工建设各类保障性住房和实施棚户区改造8015套，完工10891套，保障性安居工程建设综合评定全省第一。亭子口库区移民基本完成，实现和谐搬迁。大力推进“扶贫攻坚行动”，实施了59个整村推进项目，启动7个片区连片扶贫开发工作，6.01万人口脱贫解困。在全省率先建立十大专项救助制度，对农村五保对象等特殊困难群体的扶助力度不断加大。建成5个全国综合减灾示范社区、6个省级养老服务社会化示范社区。创新社会志愿助残“广元模式”，为6万名残疾人提供个性化服务。

　　社会大局保持和谐稳定。认真践行党的群众路线，积极推行“五建五强”群众工作法，不断加强和创新社会治理，全市综治工作获全省一等奖。群众诉求渠道进一步畅通，信访总量持续下降，化解涉稳突出问题、稳定风险评估工作分别居全省第2位、第3位，维稳工作获全省一等奖。健全大防控体系，严厉打击各类违法犯罪活动，社会治安持续好转，人民群众对治安的满意度测评全省第2名。建立健全安全生产责任体系，深入开展安全生产专项整治，安全生产综合目标考核全省第一，安全生产宣传教育工作受到国务院安办表彰。“六五”普法深入推进，全面启动省级“法治城市”创建。应急管理体制机制建设稳步推进，各类突发事件应对处置科学有力，荣获“中国智慧应急创新奖”。国防动员、民兵预备役、“双拥”工作扎实推进。

　　各位代表！过去一年的成绩来之不易，这是省委、省政府和市委正确领导的结果，是市人大、市政协和社会各界监督支持的结果，是全市人民辛勤努力的结果。在此，我代表市人民政府，向全市人民、向人大代表、政协委员，向各民主党派、工商联、无党派人士和人民团体，向驻广部队、武警官兵、政法干警和民兵预备役人员，向所有关心支持广元改革发展稳定的同志们、朋友们，表示衷心的感谢！

　　总结过去一年的工作，我们也清醒地看到还存在一些困难和问题：一是由于受宏观经济的影响，地区生产总值等部分指标未能实现预期目标。二是要保持项目投资稳定增长压力巨大，尤其是后备重大项目偏少，产业投资特别是工业、服务业投资比重偏低。三是经济总量不大与结构不优并存，传统产业优势不突出，相当部分企业和产品竞争力不强，经济持续向好发展的基础不牢固。四是城乡居民收入增长难度较大，保障和改善民生、扶贫攻坚等任务仍然艰巨。五是发展“软环境”建设还需进一步加强，政府公共服务和依法治理能力还需进一步提升，一些地方和部门还存在办事效率不高、服务意识淡薄、法纪观念不强和“中梗阻”等现象，少数干部还存在不务实、不廉洁甚至腐败等问题。这些问题，我们将高度重视，在今后的工作中认真研究、逐步解决。

2014年主要目标任务和重点工作

　　今年政府工作的总体要求是：深入贯彻党的十八大、十八届三中全会和省委十届三次、四次全会精神，全面落实市委六届七次全会和市委经济工作暨城镇化工作会议部署，以改革统领经济社会发展全局，坚持“科学发展、加快发展、追赶跨越”不动摇，按照“生态立市、工业强市、文旅兴市、统筹发展”思路，深入实施“五大发展战略”，充分释放改革动力，挖掘经济增长潜力，保持加快发展定力，全面激发市场活力，着力保障和改善民生，促进全市经济持续健康发展、社会和谐稳定。

　　主要预期目标是：地区生产总值增长10 %；地方公共财政收入增长10%；规模以上工业增加值增长12%；全社会固定资产投资增长3.5%；社会消费品零售总额增长12%；城镇居民人均可支配收入增长10%，农民人均纯收入增长12%；居民消费价格涨幅控制在3.5%左右；城镇登记失业率控制在4%以内；城镇化率提高1.7个百分点；全面完成节能减排目标任务。

　　上述预期目标和去年预期增幅相比虽有所调低，但仍是一个高于全国、全省发展速度的目标。作出这样的安排，主要基于四个方面的考虑：第一，宏观经济形势依然复杂严峻。当前，我国已全面进入经济增长换挡期、结构调整阵痛期和前期刺激政策消化期。由于国家不太可能出台大规模的经济刺激措施，因此，希望追求高速发展的外部条件和政策取向已发生重大变化。第二，我市的经济基础和基础设施的支撑能力不强，土地、资金等要素供给和资源环境约束趋紧，要实现经济的可持续发展，要以更大的决心和勇气转方式调结构，要真正把经济发展的着力点转到提高质量和效益上来，就必须审时度势，理性确定发展预期目标。第三，广元作为集盆周山区、革命老区、地震重灾区、连片贫困地区为一体的特殊地区, “发展不足、发展滞后”仍是目前面临的主要矛盾，发展仍然是解决所有问题的关键和基础，只有保持快于全国、全省合理的经济发展速度，到2020年我们才能实现与全国、全省同步建成小康社会目标。第四，从我市面临秦巴山区连片扶贫开发等特殊机遇来看，我们也有条件在未来一段时间内保持适度快于全国、全省的经济增速。因此，提出这样的经济社会发展预期目标，符合我市的阶段性发展特征，是一个有机遇、有条件，但需要全市人民付出艰辛努力才能实现的目标。

　　围绕上述要求和目标，今年将重点抓好以下工作：

　　一、全面深化改革扩大开放，着力激发加快发展活力

　　强力推进经济体制改革。今年是全面深化改革年，我们将深入推进以经济体制改革为重点的各项改革。在农村改革上，加快推进农村产权制度改革，全面完成农村土地承包经营权确权颁证工作，开展集体建设用地使用权、宅基地使用权、农村小型水利设施产权等确权登记颁证工作，深化林权配套制度改革，推进农村产权流转交易平台建设，积极稳妥推进土地承包经营权和农民住房财产权抵押、担保、转让试点，努力增加农民财产性收入。在劳动保障制度改革上，统筹推进城乡居民养老保险、基本医疗保险制度并轨，全面推行城乡居民大病保险。在财税体制改革上，完善政府预算管理体系，进一步加强BT等融资方式监管，建立健全政府性债务风险防控机制，建立事权与支出责任相适应的制度，探索乡镇基本财力保障机制试点，稳步推进“营改增”工作。在国有企业改革上，推动国有资本加快从一般性竞争领域退出，引导国有资本重点投向公共服务、关键领域等，破除各种形式的行政垄断，支持发展混合所有制经济，完善国有资产监督管理体制，深化企业内部劳动、人事、分配三项制度改革，加快完善现代企业制度。在地方金融改革上，深化农村信用社产权制度改革，推动苍溪、利州、昭化、朝天农村信用联社改组农村商业银行工作，建立完善农村产权流转中的金融服务体系，推动民间资本参与组建融资担保集团。在投融资体制改革上，深化地方政府融资平台管理改革，完善市对县（区）投入与产出相适应的配套制度。在土地制度改革上，加强城乡建设用地增减挂钩，开展废弃工矿地复垦利用试点，强化耕地占补平衡指标储备管理，改进土地报征方式，探索建立城乡统一的建设用地市场。

　　大力发展非公有制经济。坚持权利平等、机会平等、规则平等，进一步清理不利于非公有制经济发展的不合理规定，全面落实支持非公有制经济发展的各项政策措施，切实激发非公有制企业的活力和创造力。加强非公有制企业土地等要素保障，支持符合条件的民间资本设立民营银行和发展小额贷款公司、融资性担保公司等准金融机构，拓宽中小微企业融资渠道。鼓励非公有制经济参与国有企业改革，鼓励发展非公有制经济参股、控股的混合所有制企业。

　　加大开放合作力度。抓住国家对外对内开放的战略机遇期，深化全方位开放合作。按照“招大引优、集群集聚、强化履约、推进落实”的思路，在中外知名企业四川行、西博会、西交会等重大招商引资活动中创新方法、主动有为，有力推进资源转化和承接产业转移，全年引进到位市外资金366亿元以上，同口径增长10%以上。促进外贸出口，加快发展以机械电子、新材料和生物医药等产业为重点的对外加工贸易。积极参加惠民购物全川行动、川货全国行、万企出国门等技术经贸促进活动，加强境内外投资贸易交流合作。强化多层次多区域合作，推进港澳台合作，深化浙广、九广合作，扩大厅市、市校等合作，增强发展的动力与活力。

　　二、始终突出项目投资工作，力促经济平稳较快增长

　　优化投向扩大投资规模。围绕对广元长远发展具有重要支撑和强大引擎作用的方向性、引领性、带动性重大产业项目，加大工作力度，推动产业投资快速增长；引导支持企业加大技术改造和技术创新投入，争取多上快上一批市场前景好、技术水平高、就业带动强的产业项目。瞄准国家政策支持方向，围绕对全市经济社会发展具有关键性、先导性、基础性、示范性、推动性作用的重大基础设施、重大公共服务、重大民生及社会事业、重大生态环保等项目，做好向上衔接沟通工作，争取更多项目列入国、省规划，推动政府性投资稳步增长。鼓励、引导、支持民间投资，全面梳理制约和阻碍民间投资的各项规章制度，最大限度地缩小审批和核准范围、降低准入门槛，激发民间投资活力，不断扩大民间投资规模。

　　狠抓“四个一批”重大项目建设。扎实开展“项目攻坚年”活动。加快推进西成客专、上石盘电航综合枢纽工程等24个续建项目，确保完成投资147亿元以上；力促广元经济技术开发区堤防道路及配套基础设施建设、旺苍县75万吨/年铁精粉生产建设等14个项目尽快开工，确保完成投资28亿元以上；切实抓好绵万高速公路广元段、大唐广元新建电厂等26个重大项目前期工作，力争尽快通过审批；充实完善项目储备和项目库建设，建立完善动态管理机制，为广元长远发展打下坚实基础。

　　强化项目建设保障工作。加强筹资、融资工作，搭建政府、金融机构、企业多方合作平台，多渠道筹集项目资金。强化节约集约用地，严格土地用途管制，抓好征地拆迁和安置补偿。加强煤电油气的综合协调，努力保障供给。继续坚持重大项目“一名领导挂帅、一个部门主抓、一套班子落实、一个方案实施”的工作机制，建立健全来广投资企业投诉协调和法律服务机制。强化项目建设督查督办，完善项目工作目标考核机制。

　　三、深入实施创新驱动战略，进一步加快工业强市步伐

　　着力培育主导产业和重点企业。立足“5+2+1”产业体系，不断做大做强主导产业，在做大总量中调结构转方式，在扩大增量中提质增效转型升级。继续大力发展五大特色支柱产业，力争产值增长12%以上。不断壮大两大特色培育产业，力争产值增长15%以上。加快发展战略性新兴产业，力争产值增长15%以上。实施重点企业培育计划，以四川电子军工集团、长虹电子科技、娃哈哈、剑兴锂电池等企业为重点，着力培育一批主业突出、核心竞争力强、行业影响力大、带动作用好的大企业大集团。加大对中小微企业的扶持力度，力争新进规模以上企业50户。

　　切实加快产业园区和产业项目建设。切实抓好国家级广元经济技术开发区和旺苍、剑阁两个省级经济开发区的发展，加快推进苍溪县、朝天区创建省级经济开发区和利州区创建省级高新技术开发区工作，大力推进天然气综合利用工业园、081机电电子产业园等特色产业园区建设，力争园区主营业务收入增长12%以上。加强园区基础设施及配套设施建设，不断提升园区承载能力。加快亭子口水利枢纽工程3号和4号机组、川煤180万吨原煤洗选等重点项目建设，确保按期竣工投产。做好中纺60万吨油脂、兴能新材料二期等重点项目协调服务工作，确保按预定进度推进。加强对重大入园项目的前期论证和评估工作，严格项目准入条件，强化投资强度和生态环境约束，提高项目质量和效益。

　　扎实推进科技创新工作。进一步推动市校合作，加强高科技人才的培养、引进和借智引力，加快产业技术研究院、技术创新联盟、工程技术研究中心等研发平台建设，构建产学研用相结合的协同创新体系，力争形成一批重大科技成果。重视科技成果转化，加快培育高新技术企业，全年培育申报国家高新技术企业15家、省级创新型企业10家，力争高新技术产业产值增长20%以上。加大传统产业技术的改造提升力度，引导支持企业积极研发新工艺、新技术、新产品，提升产品质量。切实做好企业科技咨询和培训工作，建好科技创新服务中心和院士（专家）工作站。大力实施信息化与工业化融合工程，扎实推进“智慧广元”建设。实行市长质量奖，大力推进工业品牌战略和企业标准化建设，支持企业依托专利技术和优质产品创建国家级、省级品牌，不断提高“广元造”产品市场知名度和产销率。

　　四、深入推进“三农”工作，努力实现农民持续稳定增收

　　加快推进现代农业发展。在稳定粮食、生猪生产能力的基础上，新建现代农业园区10个、发展核心基地面积15万亩，新引进龙头企业7家、新培育农民专合组织100个。以现代农业园区综合配套，现代农业、林业、畜牧业重点县和粮经复合产业基地建设为依托，强化农业新产品、新技术、新模式的推广应用，大力发展红心猕猴桃、木本油料（核桃、油橄榄、山桐子）、茶叶烟叶、蔬菜（食用菌）、土鸡五大百亿产业，完善特色产品标准体系，推进特色农产品绿色有机化进程，提升“广元七绝”、“剑门关土鸡”品牌效应。

　　努力建设幸福美丽新村。坚持“统一规划、确立主导、整合资源、组装配套、成片成带、梯次推进”原则，实施省级新农村建设示范县、中央财政小型农田水利重点县、以工代赈新农村示范片（示范村）等项目，统筹新村聚居点建设、综合体（新型农村社区）建设、旧村落改造和传统村庄院落民居保护，完善移民库区发展规划，整体推进200个“业兴、家富、人和、村美”的新村建设。加强农田水利基础设施建设，新建和整治渠系350公里，实施土地综合整治14万亩，新建高标准农田10万亩，新建农村公路1360公里、安保设施800公里，解决8万人饮水安全问题。完成农村危旧房改造2854户，新建农村沼气服务网点75个、沼气池1.43万口。进一步规范配套村（社区）服务中心，提高农村公共服务水平。

　　切实抓好扶贫攻坚。落实各项强农惠农富农政策，从多方面帮助农民提高收入水平。认真实施秦巴山区区域发展与扶贫规划，以北部山区、边远地区、移民安置区等为重点，加大精准扶贫力度，扎实推进基础设施、特色优势产业、生态建设、能力提升、公共服务保障、微型金融服务“六大扶贫攻坚行动”。实施7个片区连片扶贫开发，启动实施整村推进项目50个，减少农村贫困人口5万人。

　　五、加快发展现代服务业，强力推进文旅兴市

　　推进文旅深度融合发展。突破性发展旅游业，实现旅游总收入140亿元、增长24.3%。创建全国旅游标准化示范市，剑门关─翠云廊创建成国家5A级旅游景区，启动剑门蜀道国家级旅游度假区建设，积极推进剑门蜀道“申遗”。精心筹办2014年四川国际文化旅游节，办好广元女儿节。大力发展文化产业，深度挖掘蜀道文化、三国文化、女皇文化、红色文化、民俗文化，加快文化产业园建设，积极培育具有较强竞争力的文化企业，丰富旅游景区文化活动，深入推进文旅深度融合发展。

　　加快建设区域性商贸物流中心。加快推进远成物流等重点项目建设，积极构建“一核两区多点”物流发展格局。继续推进万达广场等商贸综合体项目建设，加快打造老城、两江口、利州广场、万源四大商圈和特色商业街区，以广元盘龙汽车城等为重点，加快各类专业市场建设。办好秦巴山区年货节等特色节会。进一步加强城区农贸市场规划建设和管理，稳步推进农村商贸服务中心和商贸集镇建设，不断完善农村商贸流通服务体系。积极促进旅游休闲、健康养老、文化教育等服务消费，加快发展电子商务、网络服务等新兴服务业。

　　推进金融服务业发展。加快推进各类银行和金融组织发展，支持国有商业银行、农村信用社、村镇银行、小额贷款公司、农村资金互助社等金融组织更好地服务地方发展。充分利用多层次资本市场和保险、证券、基金的融资功能，扩大直接融资规模。加强投资咨询行业监管，推进金融生态环境建设。全年新增贷款70亿元，直接融资突破40亿元。

　　六、着力城乡统筹发展，有序推进新型城镇化

　　深入实施“两化”互动、城乡统筹发展战略，以“三园一区”建设为载体，继续推进“六个统筹”、“五项改革”，力争在统筹城乡综合配套改革方面取得新成效。

　　统筹优化城乡发展布局。加快完善提升城乡规划，启动广元市城市总体规划中期评估，进一步完善城镇体系规划，县城和建制镇控详规覆盖率分别达到100%、80%，完成新村规划700个以上。按照“一主两副多节点”的空间布局，向西推进三江新区建设，向北开发城北片区，向东发展大荣片区、元坝片区，向南完善万源新区，做强中心城市。推进旺苍、苍溪撤县建市工作。加大省级“百镇建设试点行动”推进力度，启动7个市级重点集镇建设，加快建设区域性中心城镇。

　　加强城镇建设和管理。按照“保续建、保急需、保重点”的原则，加快实施一批重点项目，力争陵宝二线、北二环等14个项目竣工，陵江至盘龙道路改造、万源如意湖及配套设施等23个项目开工建设。大力推进给排水、环保等基础设施建设，完善教育、医疗、文化、商业网点等公共服务设施，进一步提升城镇内在品质。围绕创建国家历史文化名城，有机融合传统和现代文化元素，提升城市文化内涵。整顿规范建筑业和房地产业市场秩序，促进其健康平稳发展。落实好城市“网格化”管理制度，不断提高城市管理的规范化、信息化、精细化水平。

　　不断创新完善新型城镇化工作体制机制。新型城镇化的推进过程是以人为核心的改革过程。按照“自愿、分类、有序”的原则，进一步落实就业、养老保险、教育、医疗卫生等配套政策，推进基本公共服务向常住人口覆盖。加强新型城乡社区建设，推进公共服务体系向农村延伸。深化户籍制度改革，全面放开城镇户籍限制，实施流动人口居住证制度，积极引导已转移到城镇就业的农村人口在城镇落户，推进农民就近就地市民化。切实抓好住房保障，各类保障性住房建设和棚户区改造不低于1万套。完善新型城镇化投融资平台，鼓励社会资本参与城市公用设施投资运营，构建多元化的资金保障机制。

　　七、加强生态文明建设，提升可持续发展能力

　　大力发展低碳经济。深入推进低碳发展战略，实施好低碳城市试点项目。大力发展水电、风电、生物质发电、天然气、农村沼气等清洁能源，加快“气化广元”进程，推进新能源示范城市创建。引导企业加快低碳技术改造，促进低碳新技术、新工艺、新设备和新材料的推广应用，着力促进产业结构低碳化。大力发展循环经济，加快发展生态农业、观光农业，继续推广测土配方施肥，推进农业持续健康发展。积极引导公众践行低碳生活理念，不断提高全民低碳发展参与度。加强与科研院校的合作，探索建立低碳统计、计量和监管体系。

　　持续优化生态环境。深入推进天然林资源保护、退耕还林等重点生态工程，加强自然保护区和森林公园、湿地公园建设，稳步提升森林覆盖率，夯实嘉陵江上游生态屏障。严守城市生态保护红线，深入推进城乡环境综合治理和爱国卫生运动，建成国家卫生城市，加快国家园林城市和省环境保护模范城市创建工作，积极开展“争做文明广元人、共建美丽广元城”教育活动，扎实抓好水环境、空气环境、固体废物和噪声污染防治，持续改善人居环境质量。坚持生物治理和工程治理相结合，强化地质灾害防治和水土流失综合治理，提高防灾减灾能力。

　　切实加强节能减排。认真执行国家政策，坚决遏制高能耗、高污染行业的过快增长，确保万元生产总值和工业增加值能耗分别下降3.2%、4%。抓好工业节能，强化建筑节能监管，大力推广绿色建筑。抓好交通、服务业、机关单位等领域节能降耗，有效推进公共机构节能。坚持环境容量总量控制，严格落实环境影响评价“三同时”制度，试行排污许可制度，加强工业污染、生活污染、农业面源污染防治。推行清洁生产，不断削减主要污染物排放总量，确保化学需氧量、氨氮、二氧化硫、氮氧化物排放量分别降低2%、1.5%、0.5%、6%。

　　八、切实保障和改善民生，加快发展社会事业

　　扎实办好民生实事。始终把保障和改善民生作为公共财政支出的主要投向，确保民生支出在公共财政支出中的比重不低于65%。不折不扣地完成就业促进、扶贫解困、教育助学、社会保障、医疗卫生、百姓安居、民生基础设施、生态环境、文化体育、菜篮子“十项民生工程”和32件重点民生实事。在扎实办好17件省定民生实事的同时，认真抓好15件市定重点民生事项：发放创业小额担保贷款7500万元、带动就业1.2万人，扶持青年创业项目20个；将参加城镇居民医保37.4万人和新农合228.97万人纳入城乡大病医疗保险；实施公益性初级卫生救护“百万培训项目”，培训20万人，孕产妇死亡率控制在25/10万以下，5岁以下儿童死亡率和婴儿死亡率控制在7‰、5‰以下，新增艾滋病检测点80个，开展“两癌”检查2.6万人；发放住房租赁补贴7550户；实施白龙湖生态环境保护项目，加大力度推动网箱饲料养殖向生态养殖转变；加强城市水环境治理，改造雨污分流管网8公里；实施主城区应急供水工程，建成2口大口井；开工建设万达中学和市示范性综合实践基地；整治背街小巷6条，建成人行过街天桥5座；实施南河南岸、北岸休闲长廊和生态修复及绿道配套工程11公里，建成全民健身路径7条；加快实施国道108线陵江至宝轮改线工程；完成则天路延伸段和则天路北段改扩建工程；续建北二环三段、四段道路及配套工程；续建广巴广陕高速公路连接线工程；续建广南高速公路万源至龙潭连接线工程。

　　深化社会事业改革发展。深入推进教育领域综合改革。大力推进城乡义务教育均衡发展，努力提升高中教育办学质量，突出职业学校产教用融合，积极支持川北幼儿师范高等专科学校等高等院校加快发展，组织实施第二轮“学前教育三年行动计划”和“特殊教育提升计划”。深化医药卫生体制改革。巩固完善基本药物制度，提高基本公共卫生服务水平；做好困难群众重特大疾病医疗救助，鼓励支持社会办医，创建全国农村中医工作先进市；完成基层卫生机构标准化建设，提高重大疾病防控和卫生应急处置能力。加快文化改革发展。推进国有经营性文化单位改制，抓好群众文艺和文艺精品创作，实施好送文化到基层“百千万”工程，丰富人民群众精神文化生活。加快推进地面数字电视建设，大力提高本地节目的覆盖率。全面做好人口计生工作，确保人口自然增长率控制在4.5‰以内。广泛开展全民健身活动，积极参加省十二届运动会，认真筹办省十三届运动会。大力推进科学技术的普及和应用，不断提升全民科学素养。做好国防动员、民兵预备役、征兵和拥军优属工作。开展好第三次全国经济普查。继续抓好国家级档案馆创建工作。认真做好审计、统计、测绘地理信息、防震减灾、地方志、工会、老龄、妇女儿童、青少年、残疾人、慈善、红十字会、气象、水文、民族宗教、外事、侨务、港澳台等工作。

　　九、依法加强社会治理，努力维护社会和谐稳定

　　加强和创新社会治理。坚持依法治市，继续推进“法治城市”创建工作。努力探索依法加强和创新社会治理体制机制，建立健全“党政统揽、系统联动，梯次推进、资源整合，创新驱动、精细服务”的工作机制，加强城乡社区建设和创新管理服务，加快社会公共服务平台建设。坚持源头治理，全面推广乡镇“综治工作中心”、“治安户长制”等建设，加强和改进流动人口、特殊人群、非公有制经济组织和社会组织的服务管理，实现网格化管理全覆盖、社会化服务零距离，努力提升社会治理水平。不断完善大调解工作体系和“三通四联两倒查”工作机制，深入推进“阳光信访”，确保信访总量不断下降。重大涉稳事项实行挂牌督办，推动稳定风险评估全覆盖，从源头上防控稳定风险。

　　努力维护公共安全。始终坚持抓好安全生产，着力完善安全生产责任体系，扎实开展“道路交通安全综合整治攻坚年”行动，继续推进煤矿、非煤矿山等重点领域的安全整治，全面落实企业主体责任，夯实安全生产基础，推动安全生产形势持续稳定好转。进一步健全社会治安防控体系，深化“平安广元”建设，严厉打击各类违法犯罪活动，不断提升人民群众安全感和满意度。建立健全食品药品安全监管运行机制、农产品质量安全监管和检测体系，切实维护人民群众“舌尖上的安全”。做好各类突发事件应急管理工作，确保防范到位、应对及时、处理妥当。加强国家安全、保密、消防和人民防空工作。

　　政府自身建设

　　各位代表，抓好政府自身建设和改革，是做好政府工作的基础。过去的一年，我们始终高度重视政府自身建设，依法清理规范行政权力，基本建成行政权力依法规范公开运行平台，深化行政审批制度改革，取消下放调整行政审批项目26项，进一步推进了政府职能转变。深入推行行政执法责任制，严格依照法定权限和程序行使权力，依法行政能力进一步提高。认真办理人大代表建议和政协委员提案，全年办理人大代表建议128件、政协委员提案281件，办结率100%，满意率分别达到100%、98%。加强各级政府政务服务中心规范化、标准化建设，政务服务体系日趋完善。加大政府信息公开力度，提高了政府工作透明度。加大发展“软环境”治理力度，62个单位、142人被问责。严格执行中央、省委和市委关于改进工作作风、密切联系群众的规定，政风行风进一步好转。认真落实“三早”预警机制，抓早、抓小、抓廉洁细胞建设，强化对行政权力的监督制约，严厉查处违纪违法行为，政府系统廉政建设得到加强，人民群众对廉政建设的满意度测评连续三年全省第1名。

　　新的一年，我们将扎实开展党的群众路线教育实践活动，按照“为民、务实、清廉”的要求，以自我革新的精神，努力建设人民满意的法治政府和服务型政府。

　　加快转变政府职能，提高服务发展能力。紧紧围绕市场在资源配置中起决定性作用转变政府职能，努力为市场主体创造更加公平有序的发展环境。推进新一轮政府机构改革，建立统一、规范的市县两级公共资源交易平台，推进工商登记制度改革，完成事业单位分类改革。进一步简政放权，深化行政审批制度改革，清理、减少行政审批事项和行政事业性收费，重点清理调整非行政许可事项。进一步完善政务服务体系，优化审批流程，提高工作效率。认真履行公共服务、市场监管、社会管理、环境保护等职责，增加公共服务供给，提高公共服务质量和水平。严格绩效管理，突出责任落实。

　　着力推进法治建设，提高依法行政能力。始终坚持用法治思维和法治方式深化改革、推动发展、化解矛盾、维护稳定。健全依法民主科学决策机制，完善行政决策程序。严格规范行政执法和自由裁量权，加强行政复议和行政调解，切实保障公民合法权益。深入推进网络问政和政务公开，强化政府信息公开，做好行政权力依法规范公开运行工作。自觉接受人大及其常委会的法律监督、工作监督和政协的民主监督，认真听取人大代表、政协委员，以及各民主党派、工商联、无党派人士和人民团体的意见和建议，主动接受社会监督。

　　切实改进工作作风，提高执行落实能力。认真执行改进工作作风、密切联系群众的若干规定。大兴调查研究之风，开展好“走基层、解难题、办实事、惠民生”和“双联”、定点扶贫等活动，努力为群众解决实际困难和问题。大力弘扬求真务实作风，反对官僚主义和形式主义。严肃行政纪律和组织纪律，深入开展“三服务”公开承诺、“百企千村万人”评效能等活动，切实提高行政效能。加大对群众反映强烈的“吃拿卡要”、“中梗阻”、“庸懒散”等问题的专项治理力度，优化发展“软环境”，确保政令畅通。

　　大力加强廉政建设，提高拒腐防变能力。认真落实党风廉政建设责任制规定和廉政准则，完善教育、预防、监督和惩治制度，加强以廉洁机关为重点的廉洁细胞和反腐倡廉基础制度机制建设。严格执行“约法三章”，厉行勤俭节约，反对铺张浪费，力戒享乐主义和奢靡之风。加强公共财政资金监督管理，严控“三公经费”和一般性支出，所有公共资金、公共资源和国有资产实现审计全覆盖。加强行政审批、工程建设、政府采购、公共资源交易等重点领域和关键环节的监管，严肃查处各类违纪违法行为，始终保持惩治腐败的高压态势，坚决纠正损害群众利益的突出问题。

　　各位代表！当前广元正处在科学发展、加快发展、追赶跨越的关键时期，时代赋予我们的使命重大而光荣。让我们在省委、省政府和市委的坚强领导下，更加自觉地肩负起历史的重托，更好地凝聚起全市人民的智慧和力量，大力弘扬“厚德行广、坚韧自强、创新开元”的新时期广元精神，为实现全面小康和建设“美丽广元、幸福家园”而努力奋斗！
